# Contribution of Doñana Wetlands to Carbon Sequestration

**DOI:** 10.1371/journal.pone.0071456

**Published:** 2013-08-19

**Authors:** Edward P. Morris, Susana Flecha, Jordi Figuerola, Eduardo Costas, Gabriel Navarro, Javier Ruiz, Pablo Rodriguez, Emma Huertas

**Affiliations:** 1 Department of Ecology and Coastal Management, Instituto de Ciencias Marinas de Andalucía (ICMAN-CSIC), Consejo Superior de Investigaciones Científicas, Puerto Real, Spain; 2 Estación Biológica de Doñana (CSIC), Sevilla, Spain; 3 Universidad Complutense de Madrid, Madrid, Spain; 4 Universidad de Murcia, Murcia, Spain; University of Yamanashi, Japan

## Abstract

Inland and transitional aquatic systems play an important role in global carbon (C) cycling. Yet, the C dynamics of wetlands and floodplains are poorly defined and field data is scarce. Air-water 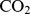 fluxes in the wetlands of Doñana Natural Area (SW Spain) were examined by measuring alkalinity, pH and other physiochemical parameters in a range of water bodies during 2010–2011. Areal fluxes were calculated and, using remote sensing, an estimate of the contribution of aquatic habitats to gaseous 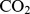 transport was derived. Semi-permanent ponds adjacent to the large Guadalquivir estuary acted as mild sinks, whilst temporal wetlands were strong sources of 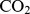 (−0.8 and 36.3 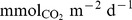). Fluxes in semi-permanent streams and ponds changed seasonally; acting as sources in spring-winter and mild sinks in autumn (16.7 and −1.2 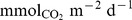). Overall, Doñana's water bodies were a net annual source of 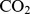 (5.2 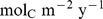). Up–scaling clarified the overwhelming contribution of seasonal flooding and allochthonous organic matter inputs in determining regional air-water gaseous 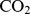 transport (13.1 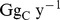). Nevertheless, this estimate is about 6 times < local marsh net primary production, suggesting the system acts as an annual net 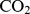 sink. Initial indications suggest longer hydroperiods may favour autochthonous C capture by phytoplankton. Direct anthropogenic impacts have reduced the hydroperiod in Doñana and this maybe exacerbated by climate change (less rainfall and more evaporation), suggesting potential for the modification of C sequestration.

## Introduction

Inland and transitional (i.e., neither fully open coastal nor enclosed or flowing freshwater) aquatic systems are hotspots for biogeochemical transformations, and in particular play an important, previously under appreciated, role in global carbon (C) cycling [1]–. Current estimates suggest that lakes, reservoirs, rivers, estuaries, ponds, streams and wetlands make a substantial contribution to global air-water C fluxes, long-term C accumulation in sediments and may play a role in regulating the climate [5]–[7].

Inland waters are estimated to emit 1.4 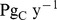 to the atmosphere, are responsible for the burial of 0.6 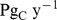 in sediments and transport 0.9 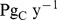 to the sea [5], [6]. Hence, the total terrestrial organic carbon (OC) imported to inland waters is in the order of 2.9 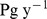, which is comparable in magnitude to the terrestrial C sink for anthropogenic emissions [1] and terrestrial net ecosystem production [5]. Lakes [8]–[10], reservoirs [11], rivers [12], estuaries [13], [14] and streams [15] make up the majority of these estimates, with each aquatic component contributing between 10 to 30% of the present total inland-transitional air-water 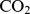 flux [5], [6]. Nevertheless, whilst it is clear that inland-transitional waters are a vital component of global C cycling, the magnitude of fluxes are still relatively poorly known in terms of the global estimated area of water bodies and the diversity of aquatic systems with good data coverage [2], [16], [17]. The potential role of very small streams, lakes and ponds, as well as wetlands and anthropogenic water bodies (such as rice paddies, farm ponds, reservoirs and drainage-irrigation networks) is still to be adequately understood [1], [2], [6]. Indeed recent studies suggest strong scale–dependence of fluxes [4], [18], reinforcing the need for detailed data coverage.

Globally smaller water bodies probably account for the majority of inland waters (lakes <1 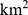 may account for >50% of the total area of all lakes [16]) and tend to have very high rates of areal 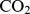 fluxes and OC burial. This is particularly the case for small agriculturally eutrophic impoundment's, which alone have recently been estimated to bury more OC each year than the oceans [2], [19]. The global area of wetlands (i.e., land surface that regularly has inundated, or saturated, conditions [20]) is about 3 times that of lakes, rivers and streams [2], [21], [22] and these diverse aquatic ecosystems also tend to have high rates of OC burial and large fluxes of green house gases (GHG, such as 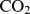, 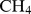 and 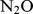) [23]–[25]. Most wetlands are only temporally flooded and contain a large (and variable) abundance of plant biomass in contact with the atmosphere, making the distinction between terrestrial and aquatic inputs of 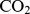 and estimation of global budgets rather difficult [5].

Getting a good grip on how these shallow aquatic systems function and their potential role in carbon cycling is particularly important considering global wetland habitat losses and the increasingly apparent effects of climate change [21], [26]. Indeed, regional differences, combined with the local effects of eutrophication and landscape changes, rather complicate predictions about how inland-transitional waters will respond to the future climate (although for some general indications see [6]). For the Mediterranean climatic region, higher minimum temperatures, more extreme high temperature events in summer and less precipitation is predicted [27]. This suggests competition for water between natural wetlands and anthropogenic activities (such as agriculture and tourism) will be exacerbated. Furthermore, as nearly all large rivers are already dammed and are under strong pressure from numerous stakeholders, ensuring the maintenance of environmental flows into Spanish wetlands may become increasingly difficult in the future [28].

As Mediterranean wetlands are characterised by a dry phase in summer to autumn, reduced runoff is likely to result in a shorter hydroperiod, as well as lower water levels and increased retention times in permanent water bodies. In consequence, smaller water bodies increasingly maintained by groundwater flows, with higher conductivity and recalitrant DOC maybe expected [6]. These will receive similar or even larger inputs of terrestrial organic matter (OM) and nutrients from anthropogenic activities, suggesting potential for increasing eutrophication. Higher temperatures will also tend to intensify the symptoms of eutrophication, on the one hand, potentially increasing autochthonous production, 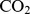 influxes [29] and OC burial [6]. On the other hand, higher rates of respiration [30] combined with alterations to community structure and increasing anoxia, may actually enhance the release of GHGs to the atmosphere [6], [31]. OM priming (or bacterial priming), were labile OC enhances the mineralization rates of more refractory OM [32]–[34] may also be an important feedback mechanism that potentially results in reduced C storage efficiency in wetlands. This maybe particularly relevant in the Mediterranean region where, because of the predictable dry phase, inputs of terrestrial and autochthonous OM have a strong temporal separation.

Climate change predictions in the Mediterranean region essentially represent a strengthening of the present seasonal trends, hence observing the seasonality of C cycling within wetlands may allow inferences to be derived about the potential effects of the future climate. Furthermore, examining water bodies with different degrees of anthropogenic influence may help understand potential interactions with eutrophication. Here we examine the spatio–temporal variation in air-water 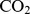 fluxes (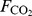) within aquatic habitats of Doñana Natural Area. By collecting a suite of physio–chemical parameters from a representative range of different water bodies, we provide insights into the mechanisms that control 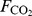 within these semi-permenant ponds, streams and temporal wetlands. We provide the first tentative estimates of air-water 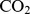 transport for the region and valuable indications about the potential role of Mediterranean, transitional wetland ecosystems in regional carbon cycling.

## Methods

### Study area

**Figure 1:**
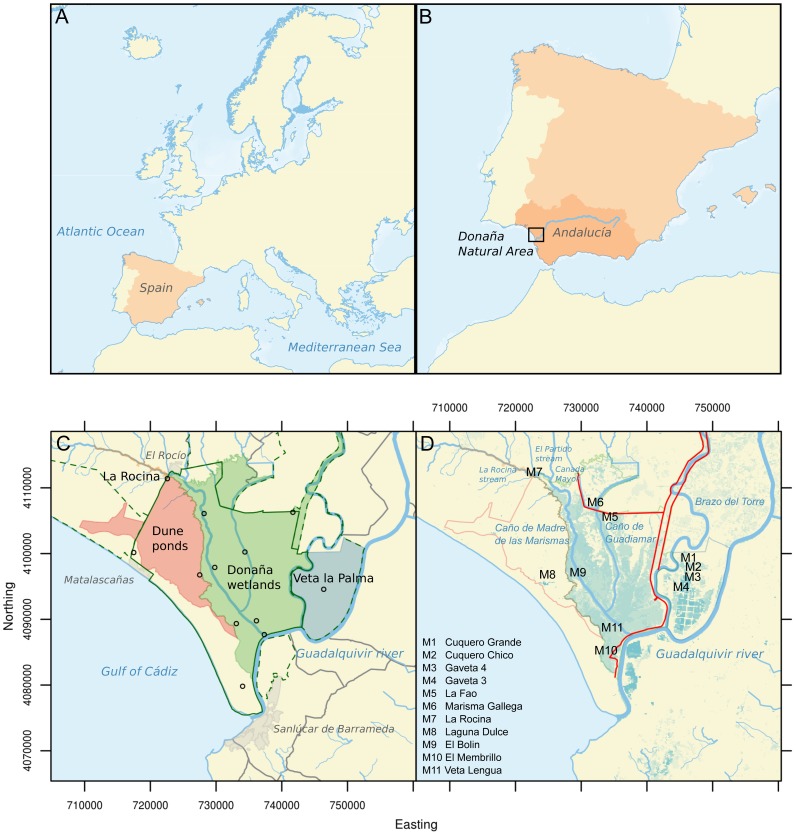
Maps showing Spain within Europe (A) and the autonomous region of Andalucía within Spain (B). Doñana Natural Area is highlighted by a black square. The Guadalquivir River is highlighted by a solid blue line. Datum: ETRS89, Projection: ETRS-LAEA. Detailed maps of the Doñana region: National and natural park limits (dark green lines), urban areas (grey polygon), positions of ICTS meterological stations (black circles) and wetland regions (coloured polygons) (C), and position of sampling sites (black text, local names given as legend), artificial structures restricting water exchange (red line) and remote sensingderived water coverage during the flood season 2010–2011 (blue shading, darker blue represents more permanent water bodies) (D). Datum: WSG84, Projection: UTM29N.

Doñana is situated on the Atlantic coast of southwestern Spain (Fig. 1, Long: −6.373, Lat: 36.932, Datum: WSG84). Covering an area of 3560 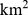 the region includes a rich variety of landforms and vegetation types representative of Mediterranean lowlands [35]. The climate is Mediterranean sub–humid with well defined seasonality; mild (average daily temperature of 9.3°C) and wet winters and dry and hot summers (25.8°C). Mean annual precipitation is about 550 mm with rainfall mostly occurring between October and March (80%) and almost absent between June and August.

The wetlands within the region have the highest degree of environmental protection in Spain (National Park status) and are one of the most emblematic protected areas in Europe with a rich biotic diversity and unique importance for wildfowl in Western Europe. Doñana National Park (ca. 54,000 ha) was designated a Biosphere Reserve by UNESCO in 1980 and a Wetland Site of International Importance by the Ramsar Convention in 1982 (entering into the Montreux Record of Ramsar sites under threat in 1990), and was declared a World Heritage Site by UNESCO in 1994 [36]. Much of the surrounding region is designated Natural (i.e., Regional) Park status with the aim of buffering human impacts on the National Park (together they are now known as the Doñana Natural Area). This status allows a larger array of traditional activities including forestry, cattle ranching, hunting, aquaculture and agriculture [35].

A diversity of aquatic systems can be found within the region including the large Guadalquivir estuary, smaller rivers/streams, such as the Guadiamar, La Rocina and El Partido, semi-permanent and temporary ponds and marshes (Fig. 1). Human interventions throughout the 20th century have drastically modified the hydrological functioning of the region, in particular reducing the total input and distribution of water within the large temporal wetlands of the National Park [28]. Water inputs from the Guadalquivir and Guadiamar rivers are very low, although restoration programs have been implemented, the main inputs of water are rainfall, the smaller streams situated in the west and ground-water.

Outside of the National Park adjacent to the Guadalquivir estuary is a privately owned wetland system with Natural Park status (Veta la Palma, ca. 11.300 ha). The area has a long history of traditional human activities and is roughly divided into 3200 ha of permanent ponds used for extensive and semi-extensive aquaculture, 3500 ha dedicated to agriculture (of which 400 ha is seasonally inundated for rice production) and 4600 ha of preserved natural marshland [37].

### Meteorological data

Rainfall (mm), air temperature (°C), atmospheric partial pressure of 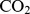 (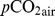, μatm) and wind speed at height *z* (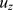, 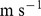) measured hourly were provided by ICTS (http://icts.ebd.csic.es) from a number of stations situated throughout the park (Fig. 1).

### Sampling design

Samples were collected from 11 water bodies (*n* = 6 to 12 per water body), selected to represent the diversity of aquatic systems within the region (Fig. 1), as part of an on going monitoring program between 2009 and 2011. Permission for field sampling was given by the director (Juan Carlos Rubio Garcia) of the Doñana Natural Area. Field studies did not involve endangered or protected species. Sites from Veta la Palma (M1:M4) are outside of the National Park boundaries and close to the Guadalquivir estuary. M1 (Cuquero Grande) and M2 (Cuquero Chico) are unmanaged temporal ponds, whereas M3 (Gaveta 4) and M4 (Gaveta 1) are used for aquaculture, with water pumped in from the estuary, and hence influenced by saline waters. In 2010–2011 water levels were maintained all season in M3, but not M4. M7 is situated at the entrance of La Rocina stream into the wetlands. M8 (Laguna Dulce) is one of the larger semi-permanent oligohaline ponds situated within a large sand dune system (the Dune ponds region). M5 (La Fao) and M6 (Marisma Galega) are semi-permanent ponds connected to the marshes and the other sites (M9:M11) are situated in temporal marshes, and thus only sampled when sufficient water was present (a minimum water depth of 0.1 m). Sampling was carried out on foot from the edge of the water body with the exact position (recorded using GPS) changing slightly depending on the distribution of water (i.e., the water level).

On each sampling occasion (approximately every 30 d) in situ water conductivity, temperature and pH were measured and water samples were collected for laboratory analysis of suspended particulate matter (SPM), chlorophyll (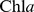), nutrients, dissolved organic matter, oxygen, pH and total alkalinity (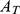). Sampling occurred during daylight hours and took 2 days, with the processing of water samples carried out in a field laboratory each evening.

### Analytical techniques

In situ water measurements of salinity, temperature and pH (National Bureau of Standards, NBS, scale) were collected with a multi-probe (YSI-6920V2, YSI Incorporated, Yellow Springs, Ohio, USA) at approximately mid-depth.

Determination of SPM as well as particulate organic matter (POM) and particulate inorganic matter (PIM) was carried out by filtering a known volume of water (pre-combusted 450°C Whatman GF/F glass fiber, diameter 47 mm). Filters were dried at 60°C for 48 h and weighed to derive SPM (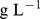), further combusted at 450°C for 5h and weighed to derive PIM and POM by difference.

Chlorophyll analysis was conducted by filtering known volumes of water (Whatman GF/F glass fiber, 0.7 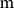 pore size), extracting in 90% acetone overnight in the dark, and measuring chlorophyll *a* concentrations using standard fluorometric methods following JGOFS protocols with a Turner Designs Model-10. The fluorometer was calibrated using a pure chlorophyll *a* standard from the cyanobacterium *Anacystis nidulans* (Sigma Chemical Company).

For inorganic nutrient analysis two 5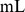 samples of filtered water (Whatman GF/F borosilicate glass fiber, 0.7 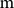 pore size) were stored at −20°C until analysis (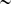
4 weeks). Concentrations of 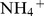, 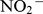, 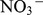, 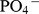 and 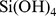 were derived following the techniques described by [38] using a 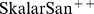 215 Continuous Flow Analyzer.

For the analysis of dissolved organic carbon (DOC) and total dissolved nitrogen (TDN) water samples were collected in situ within borosilicate vials (pre-acid-washed and-combusted, 450°C). Known water volumes were filtered (pre-combusted 450°C, Whatman GF/F borosilicate glass fiber, 0.7 μ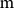 pore size) and a 24 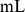 sub-sample acidified (50 μ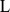 25% 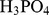, sealed and conserved at 4°C in darkness until analysis (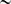
5 d). Concentrations of DOC and TDN were derived by catalytic oxidation at high temperature (720°C) and chemiluminescence, respectively using a Shimadzu TOC-VCPH analyser.

Dissolved oxygen was determined following the Winkler method. Water was carefully collected so as to avoid headspace in glass flasks of known weight. The flasks were sealed and stored in darkness, for at least 24 h until analysis. Dissolved oxygen concentrations were derived by potentiometric determination using a Metrohm 794 Titroprocessor, with an estimated error of 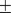
1 μ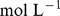.


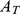
 was measured with a Metrohm 794 Titroprocessor following the method described by [39]. Water samples were collected and stored in 500 mL borosilicate bottles treated with 100 μ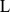 of 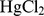 saturated aqueous solution until analysis. The accuracy of 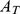 determinations was 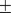
2 μ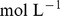 as determined from regular measurements of 2 batches (batch 85 and 89) of certified reference material (CRM, supplied by Prof. Andrew Dickson, Scripps Institution of Oceanography, La Jolla, CA, USA). Water pH (NBS scale) measurements were carried out using a Metrohm 780 pH meter equipped with a crystal electrode combination.

### Calculations

The speciation of carbon in water was calculated using co2sys.xls [40] with the dissociation constants for C and sulphate of [41] and [42], respectively. The input parameters were the measured in situ salinity, temperature (°C), atmospheric pressure (dbar), 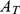 (μ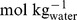), pH (NBS scale), dissolved inorganic phosphate (μ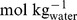) and dissolved inorganic silica (μ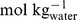). Where nutrient data was unavailable (site M3 in May 2010 and all sites in Feb 2011), the data set average of nutrient values (2.84 and 141.91 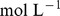 for 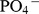 and 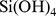, respectively) was used in calculations.

Air-water 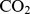 fluxes (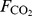, μ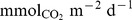) were calculated according to [43]:
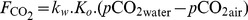
(1)where, 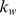 (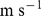) is the water–side gas transfer velocity and 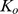 (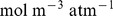) is the aqueous-phase solubility of 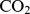 [44], [45]. 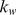 was calculated using the numerical scheme of [44]:
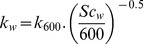
(2)where, 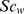 is the Schmidt number at the in situ water temperature and salinity, calculated from the diffusivity of 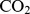, dynamic viscosity and density of water (see [45] for details) and 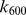 is 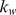 normalized to a 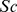 value of 600 (often quoted as typical of freshwater at 20°C). 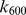 was predicted from time–ensemble averaged (1 d) horizontal wind velocity at 10 m above the surface (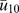, 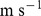) using the empirical relationship derived for lakes by [46].



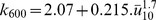
(3)

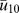
 was calculated from 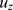 measured at nearby meteorological stations (ICTS Doñana, Fig. 1) according to [47] and spatially–averaged to give a single value for the region. We acknowledge that the empirical relationship chosen to adjust 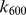 values in this study may not be ideal for representing wind–enhancement effects in this particular aquatic system (shallow water bodies with substantial variations in extent). To give an indication of the uncertainty this choice introduces, we also predicted 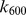 values using empirical relationships derived for small water bodies [48] (median 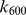 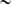
30% lower) and the global oceans [43] (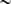
30% higher values). Daily values of water temperature, salinity, 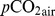 and 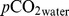, needed for calculation of 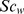 and 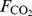, were estimated by linear interpolation of the measured monthly values. Annual areal 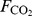 was estimated by summing daily values.

A first approximation of daily air-water 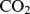 transport (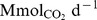) for Doñana National Park and Veta la Palma was calculated by averaging the areal 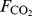 values of all sites within each wetland region (see Fig. 1) and multiplying by the surface area of water in each region respectively. Annual air-water 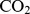 transport was calculated by summing over the year. Using the empirical relationships derived for small water bodies and global oceans to adjust 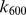 values would give annual air–water 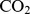 transport estimates 35% lower and 29% higher, respectively.

Water coverage was derived by remote sensing; using cloud-free Landsat TM (30 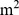 pixel size) and DEIMOS1 (22 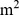) scenes collected between Nov. 2009 and March 2011. Briefly, satellite images were orthorectified against high precision airborne-photos available from the Andalucian geo-spatial infrastructure (REDIAM, http://www.juntadeandalucia.es/medioambiente/site/web/rediam, Ortofoto Digital Color de Andalucia 2008–2009). Images were radiometrically calibrated and atmospherically corrected using an image-based procedure. Finally, the multi-temporal image series was normalised using major-axis regression on a number of pseudo invariant features (such as sandy beaches, man-made structures and offshore water) selected for their very low temporal variation. Pixels were classified as water if their reflectance in the near infra–red band was less than 0.2. The Water surface area within each region was calculated by counting the number of water pixels with each region polygon (REDIAM, Humedales IHA) and multiplying by the respective satellite spatial resolution. Estimates for each day were derived by linear interpolation.

All data files required to calculate air-water 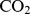 fluxes and regional transport rates are available for download from Digital.CSIC, the Institutional Repository of the Spanish National Research Council (CSIC) (http://digital.csic.es/handle/10261/77418).

### Statistics

Statistics were performed with the statistical program language R 2.15 [49]. Probability distributions of variables were examined visually and in many cases were log-normal and highly skewed. Non-parametrical Kruskal-Wallis rank sum tests (KWRS, R function; kruskal.test) and non-parametrical multiple test procedures (KWMC, Package; pgirmess, function; kruskalmc) were used to examine differences between sites [50]. Significance levels were set at 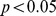. Principle components analysis (R package; FactoMineR, function; PCA, [51]) of transformed, log(*x*+1), variables, with monthly mean wind speed, total rainfall and flooding extent as a supplementary quantitative variables, was used to explore correlations. Pearson's product-moment correlation (PPMC) was used to test for significant correlations between variables (R function; cor.test).

## Results

### Meterological conditions

**Figure 2:**
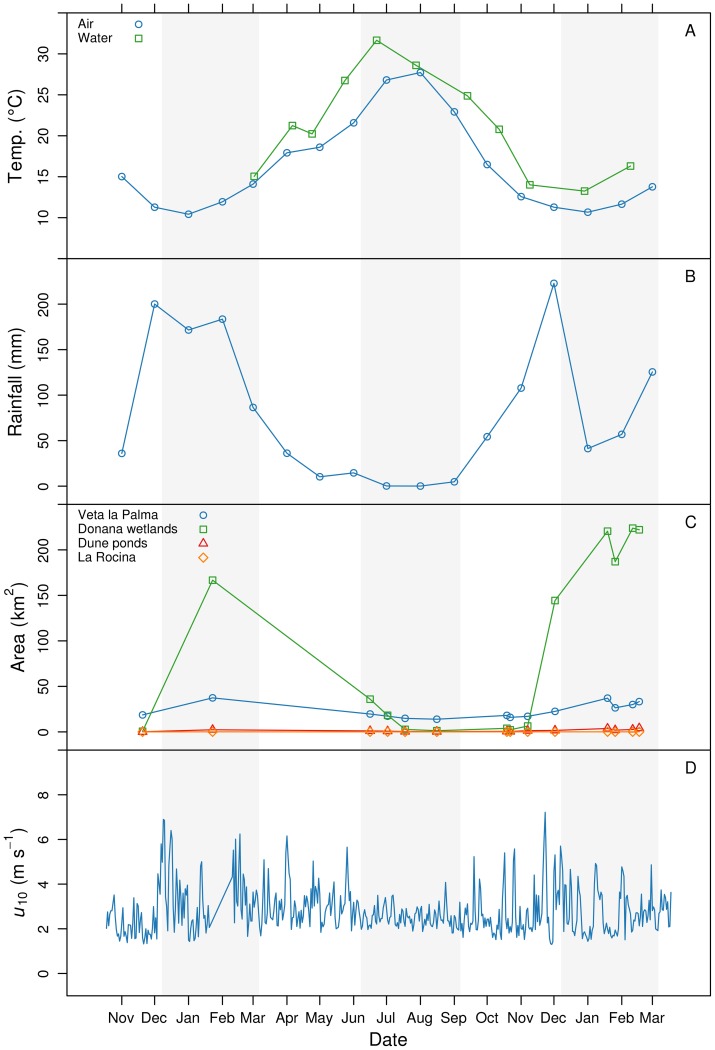
Time-series plots of air and water temperature (A), atmospheric 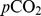 (B), monthly rainfall (C) and daily mean wind velocity at a height of 10m, 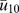 (D). Light and dark shading represents seasons.

Time-ensemble-averaged monthly air temperature ranged between a minimum of about 10.5 and a maximum of 27.7°C in January and August, respectively (Fig. 2a). Atmospheric carbon dioxide partial pressure (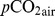) ranged between 369 and 398 μatm with a median value of 380 μatm. The lowest and highest values were observed in Feburary and August 2010, respectively. Monthly total rainfall ranged from a minimum of 0 mm in the summer of 2010 to a maximum of about 200 mm in winter 2009 and late–autumn 2010 (Fig. 2b). The wetlands of Doñana had the largest extent of water coverage reaching a maximum of 224 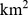 in Feb. 2011., minimum values of between 1 to 3 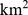 were observed in late summer–autumn (Fig. 2c). Veta la Palma had the second largest water coverage and changed relatively little throughout the study period (ranging between 14 and 37 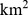). Variation in water cover in the dune ponds was more seasonal (0.1 to 4 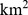), with the highest cover observed in the wet season. Because of the small size of these water bodies i.e., problems reliably detecting them with the satellite sensors used, these are probably underestimations. The same issue appeared to affect estimates of water cover in La Rocina stream, resulting in estimates of <0.1 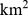. Time-ensemble-averaged (dailly) horizontal wind velocity at 10 m above the surface (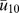) ranged between 1.3 and 7.2 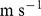, with a median value of 2.5 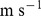 (Fig. 2d).

### Physical and chemical characteristics

Water temperature ranged between 13.6 and 31.7°C with a clear seasonal pattern that was similar at all sites (Fig. 2a). Median values of a range of other water physiochemical parameters are summarised in Table 1. Most water bodies could be considered as meso–to–eutrophic based on [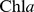] [52]. Sites in Veta la Palma (M1:M4) generally had higher median salinities (4) compared to La Rocina stream (M7) and the Dune ponds site (M8), however because of seasonal variations, most sites formed a group that ranged between oligo and mesohaline (KWMC, *p*<0.05). Significant differences between sites were also observed for most other parameters except dissolved N, 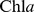 and POM (Table 1).

**Table 1:** Median chemical characteristics of the water bodies in this study.

	M1	M2	M3	M4	M5	M6	M7	M8	M9	M10	M11
Salinity	3.1^ab^	2^abc^	18.4^a^	5.5^a^	0.8^abc^	1^abc^	0.2^c^	0.5^bc^	0.5^bc^	1^abc^	1.2^abc^
O_2_ (μmol L^−1^)	308.8^ab^	331.6^ab^	270.7^ab^	342.4^ab^	450.7^ab^	337.3^a^	208.3^ab^	211.7^ab^	129.4^b^	226.5^ab^	208.2^ab^
pH (NBS)	8.2^ab^	9.1^a^	8.8^ab^	8.6^ab^	8.5^ab^	9.2^a^	8^ab^	7.7^ab^	7.6^b^	8.2^ab^	8.4^ab^
*A_T_* (μmol L^−1^)	3004.2^abc^	3760.9^ab^	3427.8^abc^	3215.5^abc^	4426.5^a^	4776.3^a^	1794.9^c^	2635.8^bc^	3143.2^abc^	4648.1^a^	5957.7^a^
DIC (μmmol L^−1^)	2.8^ab^	3.4^abc^	2.5^ab^	3.1^abc^	4.6^ac^	4.2^c^	1.8^b^	2.2^b^	3.2^abc^	4.5^c^	6^c^
PO_4_^−^ (μmol L^−1^)	0.4^a^	0.6^a^	0.4^a^	0.5^a^	6^b^	1.4^ab^	2^ab^	1.2^ab^	12.8^b^	1.6^ab^	1.4^ab^
Si(OH)_4_ (μmol L^−1^)	45.7^ab^	23.3^a^	32.7^a^	80.6^abc^	148.4^abc^	80.5^abc^	370.2^c^	241.5^bc^	217.9^abc^	160.5^abc^	168.3^bc^
NH_4_^+^ (μmol L^−1^)	2.5	2.3	2.2	2.1	1.1	1.7	4.5	2.8	3.6	1.5	1.9
NO_2_^−^ (μmol L^−1^)	1.4	0.5	0.3	1.5	0.4	0.6	0.9	0.4	0.5	0.5	0.3
NO_3_^−^ (μmol L^−1^)	1	0	0.2	0.4	0.1	0.1	6.4	0	1.3	0.2	0
DON (μmol L^−1^)	104.6^ab^	139.1^ab^	142.3^ab^	84.5^a^	69.4^a^	168.6^ab^	98.4^ab^	308.1^b^	189^ab^	101.2^ab^	108.7^ab^
DOC (μmmol L^−1^)	0.9^ab^	1.3^abc^	1.8^abc^	0.9^a^	0.9^a^	2.6^abc^	2.4^abc^	7.4^c^	4.6^bc^	1.8^abc^	2.4^abc^
Ch1*a* (μgL^−1^)	6.5	25.1	26.6	77.4	6	18.5	39.9	12.8	2.9	25.8	11.5
POM (μgL^−1^)	21.5	16	64	36.5	13	46	27	34	15	26.5	18.5
SPM (μmgL^−1^)	122.5^ab^	9.3^ab^	329^a^	143^ab^	25^b^	128^ab^	75^b^	57^b^	24.5^b^	160^ab^	81^ab^

Letters indicate significantly different groups (KWMC, 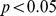).

**Figure 3:**
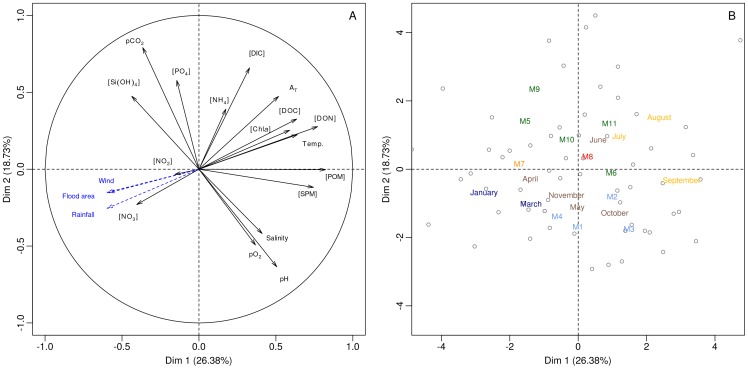
Biplots of water physical and chemical characteristics with scaling highlighting variable correlations (A) and mapping of individual samples (B). Rainfall, wind velocity and total flooded surface area are plotted as supplementary variables (blue arrows and text) on A. Text representing the centroids for each site and month are shown on B. Colours of sites and months represent regions (see Fig. 1) and seasons, respectively.

Principle component analysis revealed that the data could be summarised into 4 components that accounted for a cumalative percentage variance of 70%. In terms of briefly characterising the different water bodies only principle components 1 (PC1, 26.4% variance) and 2 (PC2, 18.7%) are discussed here. Examination of the biplots, scaled to highlight variable correlations (Fig. 3a) and individual data points (Fig. 3b), highlighted the strong correlations between many of the variables as well as general spatio-temporal trends. PC1 appeared to mainly represent seasonality; positive values were associated with higher concentrations of phytoplankton variables (POM, SPM, 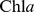 and DOM) and higher water temperatures observed in summer, whereas negative values were associated with higher [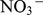], wind speed, rainfall and the extent of flooding, all winter-spring phenomena. In contrast PC2 appeared to mainly represent the differences between sites; with positive values associated with higher 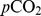, 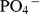, 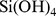 in the water bodies of the Doñana wetlands (M5, M9, M10 and M11) and negative values associated with higher Salinity, 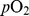 and pH in the water bodies of Veta La Palma (M1:M4). The remaining sites (M6:M8) fell between these two extremes.

### Spatio–temporal variation in dissolved 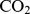


Dissolved carbon dioxide partial pressure (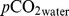) ranged between 5 and 10980μ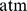 and was significantly different between sites (Kruskal-Wallis rank sum test, 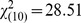, 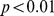) (Table 2). Median 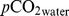 was higher in the temporal wetland site M9 (6322μ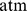) compared to the Veta La Palma site M3 (115μatm) (KWMC, *p*<0.05).

**Table 2:** Descriptive statistics for water 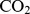 partial pressure (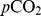,μ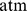) at each of the sites.

ST.ID	M1	M2	M3	M4	M5	M6	M7	M8	M9	M10	M11
Mean	758	250	201	458	3382	729	1763	2803	5382	2715	1667
Median	578^ab^	170^ab^	115^a^	302^ab^	695^ab^	115^ab^	847^ab^	2227^ab^	6322^b^	1871^ab^	1691^ab^
Standard Deviation	525	209	222	469	4373	1186	2205	3237	2942	3460	1255
Minimum	111	37	52	15	83	27	34	5	294	130	54
Maximum	1549	486	825	1455	10983	3843	7400	10230	8599	10612	3037
Range	1438	449	773	1440	10900	3816	7365	10225	8305	10482	2983
Interquartile Range	545	388	144	344	6086	912	1604	4280	2571	2330	2211
Skewness	0.4	0.2	2.3	1.3	0.9	1.8	1.7	1.1	−0.8	1.7	−0.1
Kurtosis	2.0	1.2	7.0	3.6	2.0	5.4	4.9	3.4	2.5	4.6	1.2

**Figure 4:**
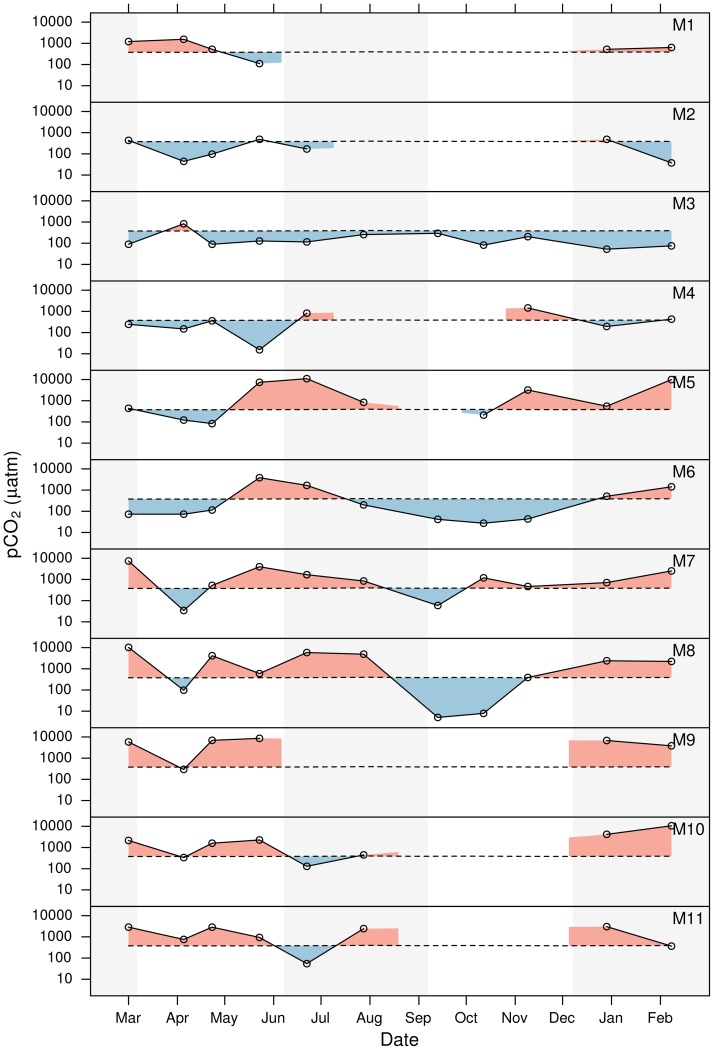
Seasonal variation in 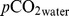 at each of the sites. The solid dark grey line is the measured 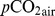. Gaps in the data represent periods when water levels were too low (<0.1 m) for sampling. Light and dark shading represents seasons.

Letters indicate significantly different groups (KWMC, 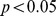).


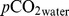
 values at site M3 were below 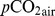 (380μ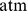) throughout the year, except for a single value of 825 μatm observed in March 2010 (Fig. 4). 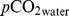 values 4 times higher than 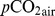 were also observed at site M1 in March, however not at the other nearby water bodies, M2 and M4, where high values were observed just before and after the drying out phase.

Within the National Park, seasonal 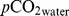 dynamics in the semi-permanent water bodies, sites M5, M6, M7 and M8 were relatively similar. All sites had very low values in early spring 2010 (about 100 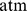) and a maximum (10983, 3843, 3975 and 5842 μ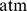 at sites M5, M6, M7 and M8, respectively) in late spring–early summer. Water levels in M5 were too low for sampling in September, however in sites M6, M7 and M8 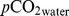 reached a seasonal minimum in autumn of 42, 59 and 5 μ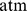, respectively. In site M8 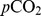 values were so low in Sept. and Oct. that 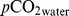 was almost completely depleted. All sites returned to a state of super–saturation with respect to atmospheric equilibrium in late winter.

In the temporal wetlands within the National Park, sites M9, M10 and M11, 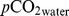 was generally highly oversaturate throughout the wet season (>2000 μatm). Relative minima of 294, 335, and 757 were observed in April at sites M9, M10 and M11, respectively. A second minimum of 130, and 54 was observed in June at sites M10 and M11, respectively. After reflooding of the wetlands in the winter of 2011, all sites had very high 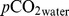 values in the order of 3000 to 10000 μ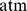.

Grouping the sites into similar types of water bodies, the mesohaline sites in Veta La Palma (M1:M4) had the lowest 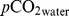 values of the region (224 μ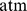) and showed relatively little seasonal variation. The temporal wetlands (M9, M10 and M11) had relatively constant and high 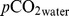 values (2358 μ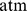) during the flood period. In contrast the semi-permanent oligiohaline sites (M5, M6, M7 and M8) had very large seasonal variations (the most extreme values observed) that could be generally characterised as low values (85 μ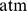) in early spring and autumn, and high values (3758 μatm) in early summer and winter.

### Water–side gas transfer velocities

**Figure 5:**
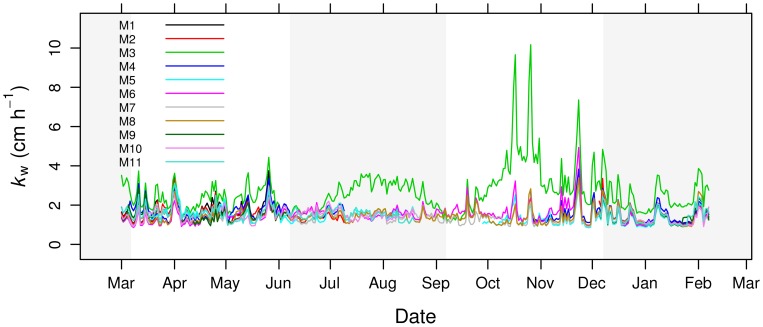
Calculated water–side gas transfer velocities (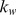) between Mar. 2010 and Feb. 2011 for each site.

Calculated gas transfer velocities (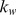) ranged between 0.85 and 10.17 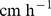 with a median value of 1.49 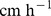. Differences in 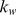 between sites were generally minor except for site M3, where high salinities (>30) resulted in substantially higher 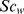 values (Fig. 5). Higher values of 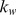 were more frequent in spring and autumn, the periods with maximum wind velocities.

### Areal air-water 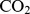 fluxes

Daily air–water 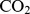 fluxes calculated for the period March 2010–2011 ranged from −19 to 197 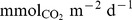 and had different statistical distributions at each site (Table 3). At most sites the mean and median values were not equal and the distributions were often positively skewed, indicating a disproportionate number of higher values (i.e., highly positive fluxes). Significant differences in fluxes were found between sites (Kruskal-Wallis rank sum test, 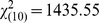, 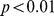). M9 had the highest median daily 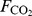, followed by a group formed by M5, M8, M10 and M11 (19 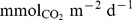) (Table 3, KWMC, *p*<0.05). The lowest median daily 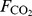 was observed in M3, followed by a group formed by M6 and M2, and slightly higher values observed at site M4 and M1.

**Table 3:** Descriptive statistics for daily areal air–water 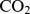 fluxes (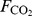, 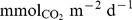) at each of the sites.

Site	M1	M2	M3	M4	M5	M6	M7	M8	M9	M10	M11
Mean	5.5	−1.5	−4.1	2.0	33.8	3.7	13.8	27.7	73.9	29.9	19.5
Median	2.4^a^	−1.3^b^	−4.1^c^	−0.7^b^	20.1^d^	−2.2^b^	6.5^e^	22.8^d^	76.6^f^	14.7^de^	17.8^d^
Standard.Deviation	8.1	2.3	4.1	6.6	39.7	11.6	19.3	28.9	35.4	39.1	14.5
Minimum	−6.9	−9.3	−19.1	−6.8	−5.3	−10.2	−4.3	−7.7	−0.8	−3.3	−2.8
Maximum	36.8	2.4	12.6	29.3	193.4	55.7	114.6	172.7	177.3	197.0	70.2
Range	43.7	11.7	31.6	36.1	198.7	66.0	118.9	180.4	178.1	200.3	73.0
Interquartile.Range	10.0	3.0	3.6	7.4	53.3	11.4	14.4	34.6	42.7	44.2	19.5
Skewness	1.3	−0.8	0.5	1.5	1.4	1.7	2.3	1.7	0.2	1.8	0.7
Kurtosis	4.4	3.1	6.0	5.1	5.0	5.4	9.2	7.5	3.1	6.0	3.3

Letters indicate significantly different groups (KWMC, 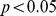.).

**Figure 6:**
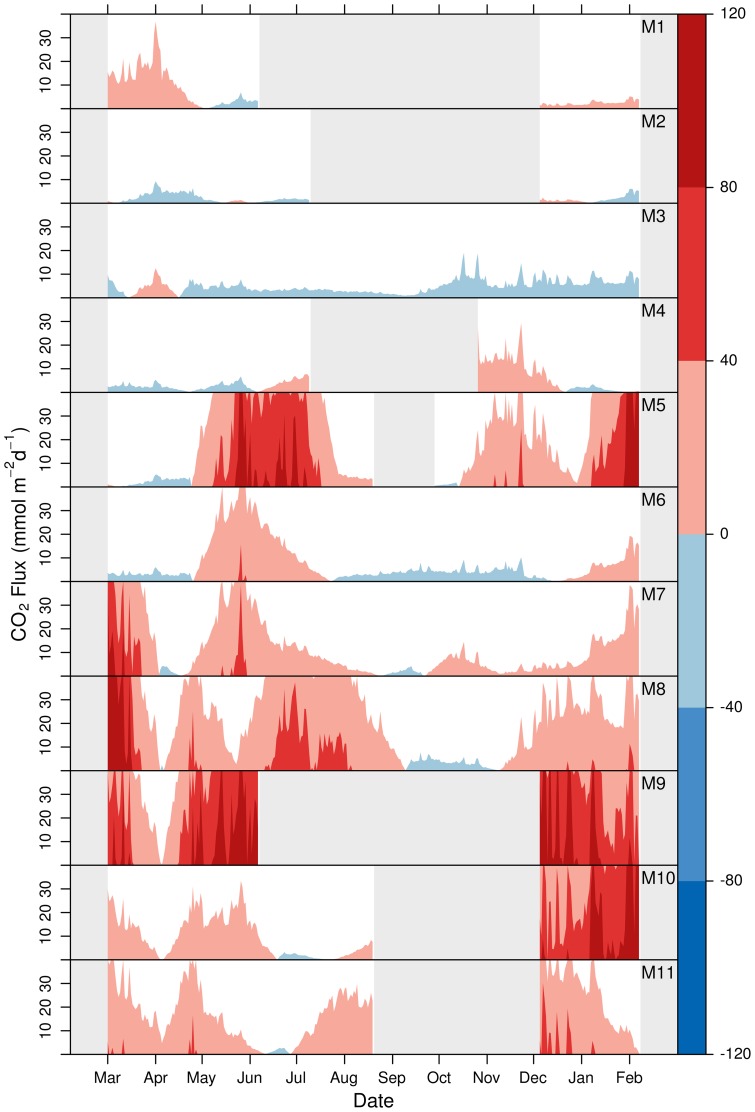
Horizon plots of 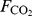 between Mar. 2010 and Feb. 2011 for each site. Colours represent the magnitude and direction of fluxes. The y axis represents one magnitude segment. Grey polygons represent no data.

The dominance of large positive values (representing release of 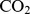 to the atmosphere) was clearly observable in the seasonal variations of 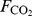 (Fig. 6, dark red represents a strong efflux of 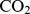). Periods with highly positive 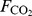 (10 to 200 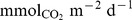), were observed in spring–early summer 2010 for all sites and at most sites (excluding M2 and M3) in the winter 2010–2011. Negative 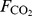 were generally of a smaller magnitude (−5 to −10 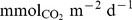) and were observed, if only for very short periods, at all sites.

### Annual areal air-water 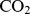 fluxes

Summing daily 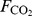 values over the year revealed that, despite having the shortest hydroperiod, site M9 was the largest source of 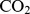 to the atmosphere (Table 4). The other wetland sites (M5, M10 and M11) also had highly positive annual 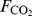 as did the dune pond site (M8) and La Rocina stream (M7). In contrast wetland site M6 had a small positive annual 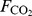, which considering the median 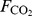 at this site was −2.2 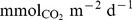, highlights the importance of short-term, but high magnitude efflux events in determining annual balances. The sites in Veta La Palma (M1:M4) were also close to equilibrium with the atmosphere and, consistent with the very low values of 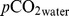 observed throughout the year, M3 had a small negative 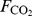, making it an annual sink for 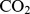.

**Table 4:** Summary of annual areal air–water 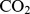 fluxes (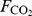) at each of the sites, range of water coverage and annual air–water C transport in each region.

Region	Site	*F*_CO2_ (mol_CO2_ m^−2^ y^−1^)	Water extent (km^2^)	C transport (Gg_C_ y^−1^)
Veta la Palma	M1	1.1	14–37	−0.06
	M2	−0.3		
	M3	−1.6		
	M4	0.5		
Doñana wetlands	M5	11.9	1–224	12.95
	M6	1.4		
	M9	13.4		
	M10	8.8		
	M11	5.1		
Dune ponds	M8	11.1	0.1–4	0.21
La Rocina	M7	6.0	0–0.1	0.004

### Regional air–water 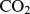 transport rates

Upscaling daily areal fluxes considering the respective surface area of water within each region (Fig. 1 and 2c) clarified the overwelming importance of the seasonal flooding of the temporal wetlands (Table 4). Because of the much larger surface area of water (4–7 times that of Veta la Palma during the flood period), the contribution of the temporal wetlands to daily air–water 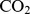 transport rates during the flood period (5–20 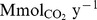) was an order of magnitude larger than the other regions. Consistent with the seasonal patterns in flooding and areal fluxes, the highest effluxes were observed in winter–spring with near zero or negative values found in summer–autumn in all regions.

Estimates of regional annual air–water C transport were 12.95, 0.21, 0.004 and −0.06 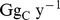 for the wetlands of Doñana National Park (M5, M6, M9:M11), Dune ponds (M8), La Rocina stream (M7) and the ponds of Veta La Palma (M1:M4), respectively. Combined together these give an estimate of total annual (2010–2011) air–water C tranport of 13.1 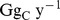.

## Discussion

Air-water 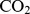 transport in the region of Doñana National Park was dominated by seasonal flooding. Strong areal effluxs of 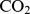 coincided with the seasonal maximums in water coverage (spring–early summer 2010 and winter 2011) resulting in high rates of net annual air–water 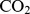 transport to the atmosphere. These fluxes were a direct consequence of the the 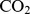 super–saturation of waters with respect to atmospheric equilibrium. This agrees with previous measurements of the ratio of gross primary production (GPP) to community respiration (CR) in the marshes of Doñana, which also suggested that flood waters were strongly hetertrophic (ratios of <0.3, [53]). The observed correlation between 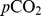 and [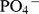] (PPMC, 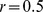, 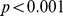, Fig. 3) suggests that this excess 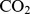 was generated by remineralisation of organic matter [54], [55].

Presumably the majority of OM in flood waters is originally derived from the vegetation within the Doñana watershed. This includes detritus washed in from the surrounding forests, scrub and agricultural land, but probably to a greater extent the local wetland vegetation (submerged, floating and emergent macrophytes) [56], [57]. There were some indications of direct nutrient inputs from anthropogenic activities in the flood waters of La Rocina stream ([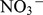] of 20 to 80 μ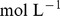), most likely chemical fertilizers derived from the surrounding agricultural activities [58]–[60], however these were undetectable within the marshes.

Autochonous pelagic primary production (including submerged macrophytes) resulting in negative air-water 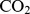 fluxes was locally relevant (Fig. 4 and 6), particularly in Veta La Palma and the water bodies that retained water during the dry season (M6, M7 and M8). High concentrations of dissolved organic matter (Table 1) were also associated with these highly productive water bodies. Very high rates of GPP have been previously reported in the semi–permanent, hyper–trophic lake Sant Olalla, situated next to Laguna Dulce (M8) [61]. Similar to our study, the highest rates of GPP were observed in spring and late summer and were accompanied by periods of very high CR meaning that annually pelagic metabolism was essentially in balance. In our study this general pattern was observed in most of the semi-permanent water bodies, however, influxes were rather small compared to the large efflux events. At a regional scale, the much larger area of water coverage within the marshes during the flood period dominated calculations of air-water 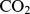 transport, overall resulting in an estimate of net 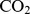 transfer from flood waters to the atmosphere.

In terms of their physiochemical properties and 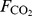 the sites in Veta la Palma, of which M3 was the only site with water all year, were clearly different compared to the National Park (Fig. 3, Table 1 and 2). Apart from being generally mesohaline, M3 also had extreme salinity variations (changing from 0 to 50 within a month) indicating management of the water levels within this pond. Indeed, because M3 is used for extensive aquaculture of commercially important fish and shrimp species, water levels are maintained by pumping in water from the Guadalquivir, which also has the side-effect of providing a suitable all year round habitat for numerous bird species [62]. These high salinity values resulted in calculated water-side gas transfer coefficients about 2 times higher than other sites in summer–autumn. Combined with the low water 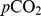 values observed throughout most of the year, this led to mild negative 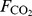, resulting in the pond acting as a mild annual sink for 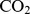.

Regular renovation of the water, lowering the water residence time, may help to reduce the build up of 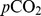 at this site. It also had among the highest concentrations of SPM and POM, but not particularly high [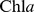], which may hint at the role of top–down grazing by meso– and macrofauna in maintaining high rates of GPP. Judging by the large quantities of commerically valuable fish and shrimp extracted from Veta La Palma, as well as the colonies of feeding birds [37], [62], a large proportion of this secondary production is presumably transferred to higher trophic levels and potentially exported from the system (some of it for human consumption).

**Figure 7:**
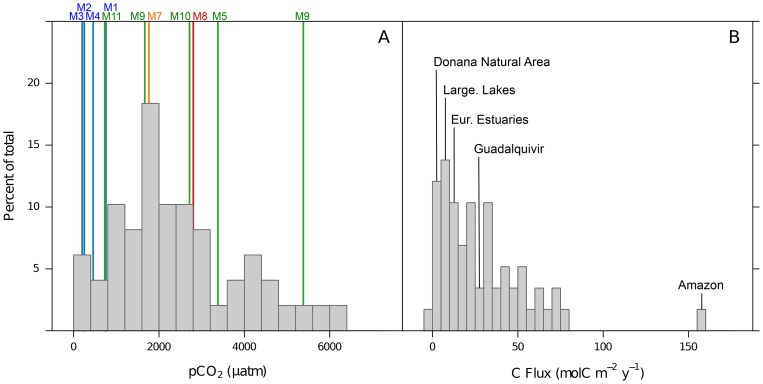
Histograms of annual mean 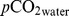 (A) and annual areal air-water 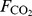 (B) of a range of inland, transitional and coastal–marine aquatic systems (citations in text). Bars represent data bins of 400 μatm (total *n* = 49) and 5 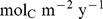 (total *n* = 58), respectively. Coloured lines and text represent the measured 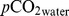 values for each site in this study. Labels highlight examples of annual areal air-water 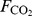 for different aquatic systems mentioned in the discussion.

Annual mean 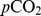 values calculated for the aquatic systems of Doñana fall within the wide range of values reported in other aquatic systems (Fig. 7a), indeed they seem to represent most of the reported range. Annual areal fluxes, however, group around the mode of literature values (Fig. 7b) [9], [63]–[65]. The mean annual flux for all aquatic systems investigated in this study (5.2 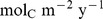) is similar to the global average for large lakes of the world (6.0 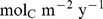, [9]) and the nearby Guadalcacin reservoir (4.6 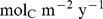, M. Morales Pers. Comm). The highest flux (13.4 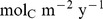), observed in the temporal wetlands, is similar to the average for European estuaries (15.8 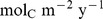, [66]), and more locally, the nearby Rio San Pedro tidal creek (16.9 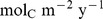, [67]) and Bornos reservoir (18.4 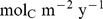, M. Morales Pers. Comm). However, it is about half the flux estimated for the Guadalquivir estuary (31.1 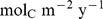, [68]) and an order of magnitude smaller than super-emitters such as the Amazon floodplain [25], [69]. Reports of negative annual air-water fluxes (i.e., net uptake of 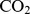) are relatively rare for inland and transitional waters, nevertheless, the lowest value observed in Veta La Palma (−1.6 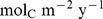) is similar to that reported for Aby Lagoon, Ivory Coast (−3.9 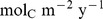, [70]) and near-shore waters of the Gulf of Cadiz (−0.4 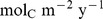, [71]).

Air–water transport is only one component of the annual net ecosystem 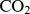 exchange (NEE) of the marshes, uptake of 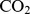 directly from the air by vascular plants and soil exchange during dry periods are not included here. Indeed, measurements of annual NEE in wetlands actually tend to suggest they are sinks for 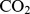 (values ranging between −2 and −15 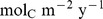, see references in [72]). Similar to our results, periods with large efflux€s are often observed in winter when plant growth is minimal, however these are offset by the high rates of net primary production (NPP) of emergent macrophytes during the growing season [73].

Whilst specific data on NEE in the Doñana marshes is not presently available (eddy covariance measurements are planned, http://icts.ebd.csic.es), potential net primary production (NPP) of the surrounding forests, marshes, heath and scrubland is estimated to be about 41, 27, 14 and 3 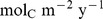 [74]–[77]. Assuming a constant NPP of marshes (27 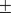 18 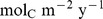) for the whole Doñana wetland region (231 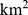 ), provides an upper estimate of potential NPP of 75
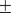
50 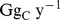, which is 2 to 10 times higher than annual aquatic air-water transport (13.1 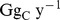 ). Although, this initial estimate of marsh NPP needs improvement and methane is likely to be quantitatively important [78], it suggests that; 1) despite a large efflux of 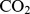 to the atmosphere during the flood period, annual NEE of the Doñana wetlands is still likely to be negative i.e., the ecosystem acts as a 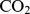 sink, and 2) the main source of C within the aquatic systems of Doñana is probably the primary production of plants within the local ecosystem.

Ponds within the managed wetland (Veta La Palma) that retained water during the dry period tended to be weak annual net 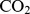 sinks. Whilst a thorough investigation of C (and other GHGs) transport is still necessary, this maybe an indication of another valuable ecosystem service provided by this particular type of low intensity aquaculture activity. For the Natural wetlands, predictions about the affects of a shortened hydroperiod [79], [80] on ecosystem 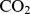 transport rates are likely to be complicated. However based on this limited dataset, we can anticipate that shorter hydroperiods may not necessarily mean less air-water transport (the site with the shortest period was the most intense emitter of 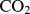 ) and at some point the NPP of the marshes will be reduced by water shortages. This suggests that the hydrological restoration program presently under way to restore the connectivity between the marsh and the Guadalquivir estuary, which should increase the hydroperiod and water renewal within the marshes, may enhance the C sequestration ecosystem service provided by the Doñana Natural Area.
